# Correction: Taming a ‘fuzzy beast’? Stakeholder perspectives on Antarctic science-policy knowledge exchange practices in New Zealand

**DOI:** 10.1371/journal.pone.0297460

**Published:** 2024-01-17

**Authors:** The PLOS ONE Staff

## Notice of republication

This article was republished on December 28, 2023, to correct some errors that were introduced during the typesetting process. The publisher apologizes for these errors. Please download this article again to view the correct version. The originally published, uncorrected article and the republished, corrected articles are provided here for reference.

## Supporting information

S1 File Originally published, uncorrected article. (PDF)

S2 File Republished, corrected article. (PDF)
