# Supplementary material for: Taming a ‘fuzzy beast’? Stakeholder perspectives on Antarctic science-policy knowledge exchange practices in New Zealand
Source: PLoS One. 2023 Nov 27;18(11):e0294063. doi: 10.1371/journal.pone.0294063 (PMC10681205; doi:10.1371/journal.pone.0294063)
Supplement: S1 File — (DOCX) [file pone.0294063.s001.docx]

**Supplementary Materials: Detailed Workshop Methods**

**Contents**

[Workshop 1 Methods 2](#_Toc131075005)

[Purpose: 2](#_Toc131075006)

[Aims: 2](#_Toc131075007)

[Workshop flow and breakout groups: 2](#_Toc131075008)

[Task 1: 3](#_Toc131075009)

[Task 2: 4](#_Toc131075010)

[Task 3: 4](#_Toc131075011)

[Data collection: 4](#_Toc131075012)

[Short-term output: workshop report 5](#_Toc131075013)

[Limitations: 5](#_Toc131075014)

[References: 6](#_Toc131075015)

[Appendix A.1. Workshop agenda 7](#_Toc131075016)

[Workshop 2 Methods 8](#_Toc131075017)

[Purpose: 8](#_Toc131075018)

[Aims: 8](#_Toc131075019)

[Workshop flow and breakout groups: 8](#_Toc131075020)

[Pre-workshop survey: 9](#_Toc131075021)

[Breakout group discussion 1: Rapid assessment of the state of Antarctic science-policy engagement in New Zealand 9](#_Toc131075022)

[Breakout group discussion 2: Analysis of strengths, weaknesses, opportunities and threats/risks (SWOT analysis) 10](#_Toc131075023)

[Plenary discussions: 11](#_Toc131075024)

[Data collection: 11](#_Toc131075025)

[Short-term output: workshop report 11](#_Toc131075026)

[Limitations: 11](#_Toc131075027)

[References: 11](#_Toc131075028)

[Appendix B.1. Workshop Agenda. 13](#_Toc131075029)

[Appendix B.2. Pre-workshop survey questions 14](#_Toc131075030)

[Appendix B.3. Google document for breakout group discussion 1. 20](#_Toc131075031)

[Appendix B.4. Google document for breakout group discussion 2. 21](#_Toc131075032)

[Appendix B.5. Scoring guide 22](#_Toc131075033)

# Workshop 1 Methods

## Purpose:

To develop a shared understanding of the Antarctic science-policy interface in the New Zealand context and identify opportunities and barriers for achieving successful science-policy interactions.

## Aims:

- Identify the key elements that facilitate Antarctic science-policy interactions in New Zealand drawing on workshop participants’ individual experience and expertise;
- Assess which of these elements are currently working well (i.e., are contributing to successful KE), and conversely, not working well (i.e., are hindering successful KE), and explore the reasons why; and,
- Identify the barriers, gaps and opportunities for improving science-policy interactions in the future.

It is important to note that the aim was not to explicitly define or explore the notion of ‘success’. For this reason, a definition for ‘success’ was provided to the participants towards the beginning of the workshop (see below).

## Workshop flow and breakout groups:

Among the workshop agenda items (see Appendix A.1.), the breakout group discussions^[[1]](#footnote-1)^ were the primary workshop activity. The research team pre-assigned all 64 workshop participants (see full article) into 12 smaller breakout groups (5-7 participants per group). Participants were pre-assigned to groups to balance expertise, gender, and career stages to enable networking opportunities and maximise the diversity of perspectives per group. Research has shown that addressing a problem from diverse perspectives results in more innovative solutions and outcomes (Hofstra et al. 2020).

Each breakout group had a volunteer facilitator. Facilitators were colleagues of the research team (i.e. not the research team themselves). Breakout group facilitation involved two key responsibilities: a) to help capture key information, and b) to ensure that all participants had the opportunity to express their views equally. The facilitators were active participants and contributed to the group discussions.

Participants were given two hours to complete three tasks (Figure 1) within their assigned groups. The research team prompted the groups to move through the tasks in chronological order, while also acknowledging that the tasks were inherently multi-directional and overlapping.


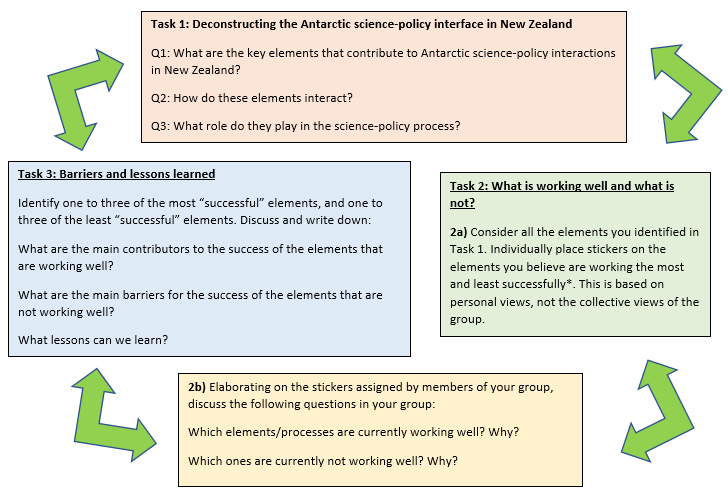


Figure 1. The three main workshop tasks.

## Task 1:

Participants were asked to discuss and answer three questions among their groups:

1. What are the key elements that contribute to Antarctic science-policy interactions in New Zealand?

2. How do these elements interact?

3. What role do they play in the science-policy process?

To help stimulate discussions during task 1, participants were given seven categories to consider in the context of the questions asked. These categories included:

- Government institutions
- Non-governmental institutions
- Social and cultural factors
- International organisations
- Intrinsic factors
- Incentive structures
- Science-advisory processes

The group facilitator assigned a note-taker whose role it was to capture the key points of the discussions using the materials provided (paper, pens, post-it notes etc).

## Task 2:

Task 2 asked participants to reflect on the elements that they had identified as having an influence on Antarctic science-policy interactions in New Zealand. They were asked to individually rate the various elements in terms of “success”.

Due to time constraints, it was not feasible to explore the notion of ‘success’ as we anticipated that this would take considerable time. In fact, given how contentious a notion success is in the science-policy research space (e.g., see Karcher et al., 2021), we considered it as essential to provide participants with a definition for ‘success’ as a starting point and to avoid lengthy discussions on this topic (noting that this is worthy of further scholarly attention). KE practices, or elements that influence KE practices, were considered to be ‘successful’ or working well if they contributed to, rather than hindered, “social processes which encompass relations between scientists and other actors in the policy process, and which allow for exchanges, co-evolution, and joint construction of knowledge with the aim of enriching decision-making” (van den Hove, 2007:807).

We note that this conceptualisation did not originally aim to define KE success. Rather, van den Hove (2007) provided this definition to describe science-policy interfaces more generally. However, it is our view that while useful, this definition is more aspirational than it is realistic i.e., KE can be far messier and complex in reality. We therefore adopted this particular definition as a way to help participants think about how KE practices *may* look like if deemed successful.

We also note that Karcher et al. (2021) have since provided the following definition for KE success: “respectfully conducted, partner-relevant research that is accessible, understandable, shared, and used, enabled by good knowledge exchange products, - processes, and social outcomes (e.g. creating networks, mutual understanding, social learning, and trust building), with the potential to contribute to changes in policy and demonstrable societal impact” (p. 214).

For Task 2A, participants were given three colours of stickers to individually rate each of the elements that they had identified during Task 1:

Green stickers = working well/‘successful’ elements i.e. the participant felt happy with this element

Red stickers = not working well/‘unsuccessful’ elements that require change or improvement i.e. the participant felt unhappy with this element

Yellow stickers = for those elements where participants felt they did not know enough to make an assessment, or they did not understand the role of this element in facilitating science-policy interactions

For Task 2B, participants were asked to reflect on the placement of stickers by their individual groups members and discuss the reasons for their decisions. This included reflecting on where the group had similar or divergent views about the function of the various elements identified.

## Task 3:

Participants were asked to choose several of the most and least successful elements (i.e., those that had received a lot of red or green stickers) and address the Task 3 questions as shown in Figure 1.

## Data collection:

Two types of data were obtained:

1. Written – all notes written down by participants (including stickers); and,
2. Verbal (Audio) – ten out of twelve group discussions were recorded on the phones of the facilitators.

All data were anonymised for the purposes of the analysis using codes that did not resemble the personal identity of the participants.

## Short-term output: workshop report

Following the workshop, the research team undertook a preliminary assessment of the high-level findings from the workshop and drafted a report to disseminate among the workshop participants for feedback. The report provided descriptive topic summaries (Braun & Clarke, 2022) of workshop discussions as opposed to the situated themes that we have since reported in our main article. The purpose of this exercise was to facilitate ongoing dialogue with the participants and to provide them with an opportunity to shape the outcomes of the research on more than one occasion. Only one participant provided feedback on the report. The report is accessible on the Antarctic Science Platform website here: [Workshop Report_NG2 MC2 (d1e7mq055r7tid.cloudfront.net)](https://d1e7mq055r7tid.cloudfront.net/knowledge-hub/ASP-003-Workshop-Report_final.pdf) (Accessed 15 March 2023).

## Limitations:

Given the complex nature of science-policy interfaces, we provided the participants with additional prompts and definitions to help frame their thinking on the issue. For example, we provided participants with a definition for KE ‘success’. Likewise, we provided specific categories for participants to discuss during the workshop to facilitate conversations. Although we told participants to use the categories as a thinking aid and that they were non-exhaustive, we observed the participants using the categories vigilantly to structure their thinking (e.g., see the jottings of one breakout group in Figure 2 below). While we encouraged groups to add new categories, only one group identified a novel category, which they named “educational institutions”.

It therefore possible that the early identification and provision of broad categories and a definition for ‘success’ served to narrow the workshop discussions and later bias the analytic process. To maximise the sincerity and rigor of our work, we spent significant time reflecting on how these predilections may have influenced the data collected and our interactions with it, concluding that the choices we made were unavoidable in the context of facilitating rich discussions about a complex topic in a short space of time.


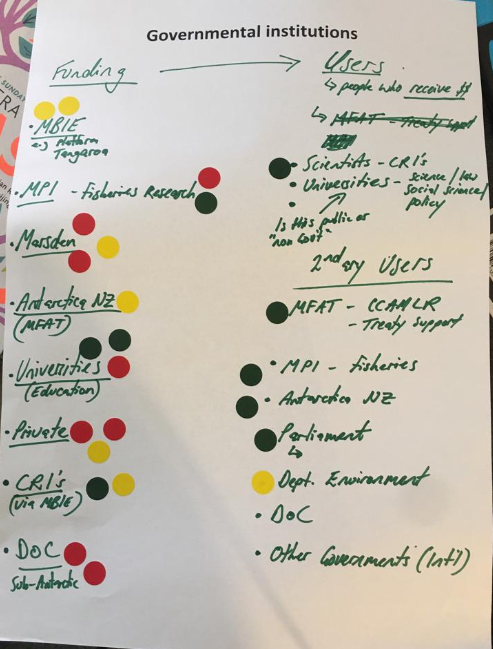


Figure 2. One breakout group’s written material relating to the category ‘Governmental Institutions’.

## References:

Braun, V., & Clarke, V. (2022). *Thematic Analysis. A practical guide.* Sage Publications Ltd.

Caretta, M. A., & Vacchelli, E. (2015). Re-thinking the boundaries of the focus group: A reflexive analysis on the use and legitimacy of group methodologies in qualitative research. *Sociological Research Online*, *20*(4), 58-70. 10.5153/sro.3812

Hofstra, B., Kulkarni, V. V, Munoz-Najar Galvez, S., He, B., Jurafsky, D., & Mcfarland, D. A. (2020). The Diversity-Innovation Paradox in Science. *Proceedings of the National Academy of Sciences*, *117*(17), 9284–9291. <https://doi.org/10.1073/pnas.1915378117/-/DCSupplemental>

Karcher, D. B., Cvitanovic, C., Colvin, R. M., van Putten, I. E., & Reed, M. S. (2021). Is this what success looks like? Mismatches between the aims, claims, and evidence used to demonstrate impact from knowledge exchange processes at the interface of environmental science and policy. *Environmental Science and Policy*, *125*, 202–218. <https://doi.org/10.1016/j.envsci.2021.08.012>

van den Hove, S. (2007). A rationale for science-policy interfaces. *Futures*, *39*(7), 807–826. <https://doi.org/10.1016/j.futures.2006.12.004>

## Appendix A.1. Workshop agenda

| 10:00 – 10:05 | Welcome and karakia |
| --- | --- |
| 10:05 – 10:15 | Context setting presentation on the Antarctic science-policy interface.  Workshop outline and instructions |
| 10:15 – 10:40 | Task 1: Deconstructing the Antarctic science-policy interface |
| 10:40 – 10:45 | Task 2a: Individual assessment |
| 10:45 – 11:00 | Task 2b: Breakout group discussions on the individual ratings |
| 11:00 – 11:10 | Coffee break |
| 11:10 – 11:50 | Task 3: Identifying gaps and opportunities |
| 11:50 – 12:00 | Workshop close and karakia |

# Workshop 2 Methods

## Purpose:

The purpose of the workshop was to bring together New Zealand’s Antarctic policy and decision-making community to explore the current state of Antarctic science-policy interactions and identify any potential new or improved ways of working.

## Aims:

- Examine the current level and quality of engagement between New Zealand’s Antarctic decision-makers and the Antarctic research community to inform both New Zealand and Antarctic policy and governance; and
- Identify processes or mechanisms that may help to facilitate the engagement between Antarctic research and policy communities.

## Workshop flow and breakout groups:

The online workshop opened with a round of introductions and preliminary remarks by the research team before Antarctica New Zealand’s Chief Scientific Advisor, gave an invited context-setting presentation on a) New Zealand’s Antarctic research outputs, and b) potential gaps and opportunities for the future of Antarctic science-policy interactions in New Zealand. See Appendix B.1. for the workshop agenda.

Participants were then invited to share their opinions, thoughts, and experiences on two broad discussion topics. For each discussion topic, participants were sent into breakout groups via the breakout room function on Zoom. There were 2-5 participants per breakout group, resulting in 5 breakout groups in total. After each breakout group discussion, the workshop participants came back together into the main Zoom room to participate in a plenary session on the discussion topic. Figure 1 illustrates the workshop flow.

The research team divided participants as evenly as possible into breakout rooms so that each breakout group comprised of a mix of individuals from different institutions/organisations. This was not however possible in some instances, for example, three individuals from the Ministry of Foreign Affairs and Trade joined the call from a shared meeting room and it was not possible to separate them into different breakout groups.


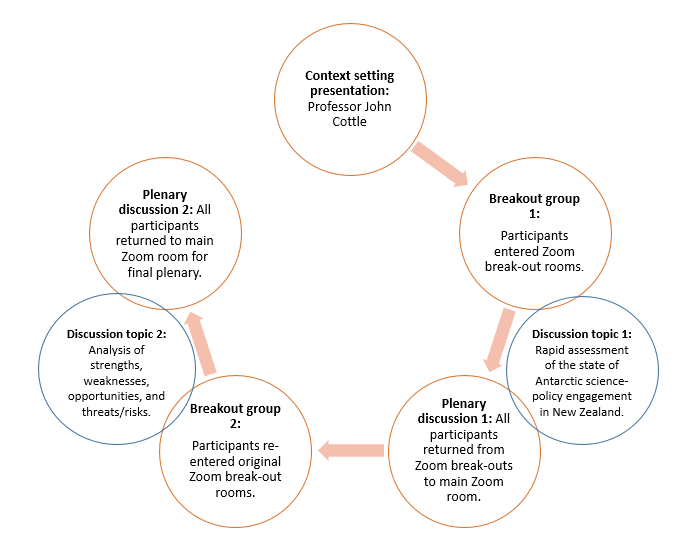


Figure 1. The workshop 2 activities.

## Pre-workshop survey:

One week prior to the workshop, the research team sent a short online survey (via Qualtrics) to participants via email, which they completed before arriving at the workshop on Zoom (see Appendix B.2.). The purpose of the survey was to: (a) gain preliminary information about the participants’ views on Antarctic science-policy interactions in New Zealand, (b) gain a sense of the extent to which they or their organizations were engaged with Antarctic research and knowledge exchange practices and, (c) get a gauge on the diversity of our participants with regard to their organizational affiliations. This information was used to inform the development of the workshop tasks.

## Breakout group discussion 1: Rapid assessment of the state of Antarctic science-policy engagement in New Zealand

For the first breakout group discussion, participants were allocated to one of the five Zoom breakout rooms to undertake a rapid assessment of the state of Antarctic science-policy engagement in New Zealand. Each of the Zoom rooms was provided with access to its own version of the same Google Document that included seven ‘influencing factors’ to rate and discuss (see appendix B.3.).

Table 1 provides further detail on the influencing factors, which included communication, transparency, incentives, timelines, trust, diversity and inclusivity, leadership and ‘other’ (optional). The influencing factors comprised seven topics that were identified by the research team during a preliminary analysis of the workshop 1 dataset.

The seven influencing factors were elements that workshop 1 participants perceived as having the potential to contribute to the success (or complexity) of Antarctic science-policy interactions. For example, how researchers and policymakers *communicate* is likely to influence knowledge exchange.

Table 1. The seven 'influencing factors' (informed by the preliminary findings from workshop 1) upon which workshop 2 participants were to base their rapid assessments, including the guiding questions and statements that were provided to help stimulate thinking.

| **Influencing Factor** | **Prompt** |
| --- | --- |
| Communication | How is information being conveyed between the involved parties and how well is this working? |
| Transparency | How clear is the process of science-policy engagement to all involved parties? |
| Incentives | How do you rate the 'incentive' landscape at all levels of science-policy engagement? What incentives exist? |
| Timelines | How well aligned are research and policy timelines? |
| Trust | Assess the level of 'trust' in the process of engagement, the parties/people involved, as well as trust in the information communicated. |
| Diversity and inclusivity | How inclusive are science-policy dialogues in taking into account a broad range of perspectives? Who is currently involved? |
| Leadership | How well is the science-policy process led and driven by engaged entities or individuals? Who are these entities and individuals? |
| Other (optional) | Can you think of any other influencing factors? |

Using a modified Delphi approach, participants were asked to rate each factor individually (1 = very poor; 3 = average; 5 = excellent) in terms of how well they thought the factor was currently working or operating at the Antarctic science-policy interface.

After the participants had provided an individual rating for each factor, they discussed their ratings among their breakout groups, including the reasoning behind their decisions. For each factor, they then agreed on a final breakout group rating.

Lastly, each breakout group gave an overall score for how they rated Antarctic science-policy interactions in New Zealand *in general* (i.e., when taking all influencing factors into account). To help with their overall assessment, the groups were provided with a rating scale/scoring guide to offer a frame of reference (see Appendix B.5. for scoring guide). Breakout groups were asked to record all key discussion points in the Google Documents and it was these qualitative data that were used for the analysis. Although participants were asked to provide scores at various points in time, the scoring exercises were used as a way to stimulate discussion rather than to produce quantitative data for further analysis.

## Breakout group discussion 2: Analysis of strengths, weaknesses, opportunities and threats/risks (SWOT analysis)

For the second breakout group discussion, participants broke out into the same Zoom rooms to undertake a SWOT analysis. They were asked to identify the strengths, weaknesses, opportunities, and threats/risks for New Zealand Antarctic science-policy interactions, now and into the future. The breakout groups were each provided with a new Google Document that provided prompts to help guide the SWOT analysis (see Appendix B.4.). Breakout groups were asked to record all key discussion points in the Google Documents.

## Plenary discussions:

After each breakout group discussion, the groups were invited back into the main Zoom room for plenary. In the first plenary discussion, groups reported back on their scores and the reasons behind their choices and were encouraged to challenge each other’s perspectives. In the second plenary session, breakout groups shared the results of their SWOT analyses and were again encouraged to question each other and challenge the viewpoints put forth.

## Data collection:

Two types of data were obtained:

1. Written – all notes recorded by the breakout groups in the Google Forms provided; and,

2. Verbal (Audio) – all plenary session discussions were recorded for transcription purposes (breakout group discussions were not recorded to encourage open and honest discussions).

All data were anonymised for the purposes of the analysis using codes that did not resemble the personal identity of the participants.

## Short-term output: workshop report

Following the workshop, the research team undertook a preliminary assessment of the high-level findings from the workshop and drafted a report to disseminate among the workshop participants for feedback. The report provided descriptive topic summaries (Braun & Clarke, 2022) of workshop discussions as opposed to the situated themes that we have since reported in our main article. The purpose of this exercise was to facilitate ongoing dialogue with the participants and to provide them with an opportunity to shape the outcomes of the research on more than one occasion. We did not receive any feedback on the report. The report is accessible on the Antarctic Science Platform website here: [ASP-04 Workshop Report FINAL3.0 (d1e7mq055r7tid.cloudfront.net)](https://d1e7mq055r7tid.cloudfront.net/knowledge-hub/ASP-Stakeholder-Workshop-Report-FINAL.pdf) (Accessed 15 March 2023).

## Limitations:

Given the complex nature of science-policy interfaces, we provided participants at both workshops with additional prompts and definitions to help frame their thinking on the issue. For example, as we identified in the methods for workshop 1, we provided a definition of ‘success’ science-policy interactions. Offering such prompts framed and thus limited the discussions to a certain extent, possibly restricting truly divergent thinking.

Likewise, we provided specific elements or categories for participants to discuss during both workshops to facilitate conversations. We also used topics that arose in workshop 1 to stimulate discussions in workshop 2. It therefore possible that the early identification and provision of broad categories and definitions, as well as the use of the first workshop’s findings for the second, was too deterministic and narrowed the workshop discussions somewhat.

To address these limitations and add to the sincerity and rigor of our work, we spent significant time reflecting on how these predilections may have influenced the data collected and our interactions with the data, concluding that the choices we made were unavoidable in the context of facilitating rich discussions about a complex topic in a short space of time.

## References:

Braun, V., & Clarke, V. (2022). *Thematic Analysis: A Practical Guide.* Sage Publications Limited.

Kahneman, D. (2021). *Noise: A Flaw in Human Judgement.* Harpercollins Publishers.

## Appendix B.1. Workshop Agenda.

1. Welcome and introductions
2. Context-setting presentation by Antarctica New Zealand’s Chief Scientific Advisor, Professor John Cottle
3. Breakout session 1 – exploring the current state of engagement between New Zealand’s Antarctic decision-makers and the Antarctic research community

Screen break

1. Plenary – discussion and reflections on breakout session 1
2. Breakout session 2 – identifying any (new) processes or mechanisms that could help to facilitate the engagement between Antarctic research and policy communities

Screen break

1. Plenary – discussion and reflections on breakout session 2
2. Next steps and close

## Appendix B.2. Pre-workshop survey questions

Antarctic stakeholder workshop

Start of Block: Default Question Block

Q1 Is Antarctic research relevant to your work or your organisation?

- Yes (1)
- No (2)

Skip To: Q2 If Is Antarctic research relevant to your work or your organisation? = Yes

Skip To: Q5 If Is Antarctic research relevant to your work or your organisation? = No

Q2 Please specify what aspects of Antarctic research are relevant and why.

________________________________________________________________

________________________________________________________________

________________________________________________________________

________________________________________________________________

________________________________________________________________

|  |  |
| --- | --- |

Q3 Please describe your or your organisation's engagement with the Antarctic research community. 


When answering this question you might want to think about the frequency of engagement, type of engagement (e.g., email, in-person, web searches, phone calls, etc.), the extent to which you or your organisation draw on Antarctic research, and the extent to which the research meets your or organisation's needs, etc.

________________________________________________________________

________________________________________________________________

________________________________________________________________

________________________________________________________________

________________________________________________________________

Q4 What internal and external factors impact your, or your organisation's, engagement with the Antarctic research community (both positively and negatively)?

________________________________________________________________

________________________________________________________________

________________________________________________________________

________________________________________________________________

Q5 How important are each of the following in defining the success of Antarctic science-policy engagement?

|  | Not at all important (1) | Slightly important (2) | Moderately important (3) | Very important (4) | Extremely important (5) | Don't know (6) |
| --- | --- | --- | --- | --- | --- | --- |
| Antarctic scientists and policy-makers co-produce research or policy. (1) |  |  |  |  |  |  |
| New policy is informed by Antarctic research. (2) |  |  |  |  |  |  |
| Policy that is informed by Antarctic research results in notable positive outcomes. (3) |  |  |  |  |  |  |
| The current level of Antarctic science-policy engagement is maintained. (4) |  |  |  |  |  |  |
| Researchers and policymakers have regular and ongoing dialogues, irrespective of research or policy outcomes. (5) |  |  |  |  |  |  |
| Institutional change results, (e.g., a new committee, process, or mechanism is developed to facilitate Antarctic science-policy interactions at the institutional level). (6) |  |  |  |  |  |  |
| Antarctic researchers and policymakers operate independently, and knowledge is shared asynchronously (e.g., through reports, and other documents/media). (7) |  |  |  |  |  |  |
| Antarctic research funding is aligned with policy strategies. (8) |  |  |  |  |  |  |
| Other (please specify) (9) |  |  |  |  |  |  |
| Other (please specify) (10) |  |  |  |  |  |  |
| Other (please specify) (11) |  |  |  |  |  |  |

Q6 Please share with us any further comments on Antarctic science-policy engagement:

________________________________________________________________

________________________________________________________________

________________________________________________________________

________________________________________________________________

________________________________________________________________

| Page Break |  |
| --- | --- |

Q7 Please indicate your affiliation (select as many as appropriate).

- Antarctica New Zealand (1)
- Ministry of Foreign Affairs and Trade – Manatū Aorere (2)
- New Zealand Defense Force – Te Ope Kātua O Aotearoa (3)
- Ministry for the Environment – Manatū Mō Te Taiao (4)
- Ministry for Primary Industries – Manatū Ahu Matua (5)
- Ministry of Business, Innovation and Employment – Hīkina Whakatutuki (6)
- Department of Conservation – Te Papa Atawhai (7)
- Land Information New Zealand – Toitū Te Whenua (8)
- Maritime New Zealand – Nō te rere moana Aotearoa (9)
- Christchurch Antarctic Office (10)
- Environmental Protection Agency – Te Mana Rauhī Taiao (11)
- Office of the Prime Minister’s Chief Science Advisor – Kaitohutohu Mātanga Pūtaiao Matua ki te Pirimia (12)
- Parliamentary Commissioner for the Environment – Te Kaitiaki Taiao a Te Whare Pāremata (13)
- Royal Society Committee on Antarctic Science (14)
- Department of the Prime Minister and Cabinet – Te Tari o te Pirimia me te Komiti Matua (15)
- New Zealand Customs Service – Te Mana Ārai o Aotearoa (16)
- Climate Change Commission – He Pou a Rangi (17)
- Local or regional council (please specify) (18) __________________________________________________
- Other (19) __________________________________________________

End of Block: Default Question Block

## Appendix B.3. Google document for breakout group discussion 1.


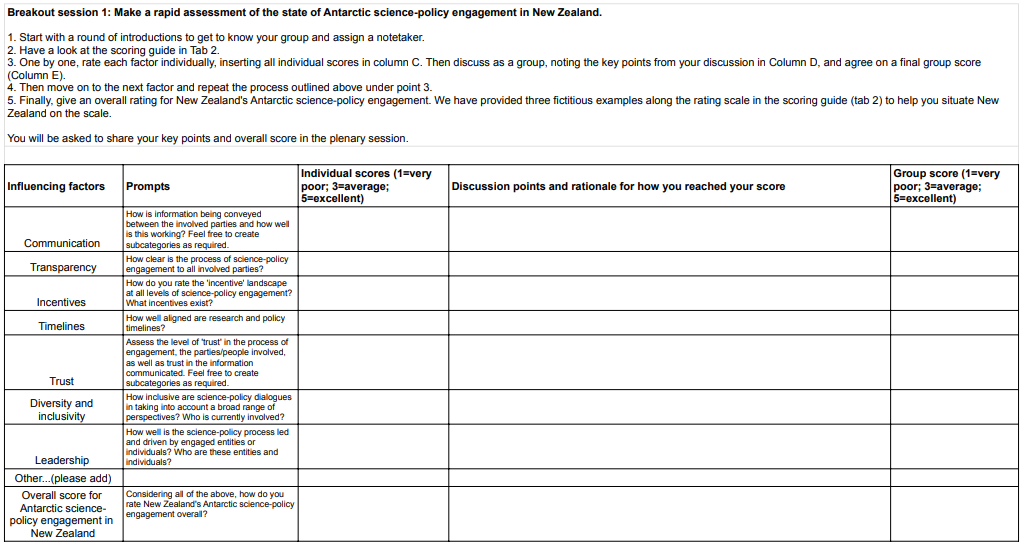


## Appendix B.4. Google document for breakout group discussion 2.

Building on your discussions in breakout session 1, what are the strengths, weaknesses, opportunities, and threats/risks regarding Antarctic science-policy engagement in New Zealand, now and into the future?

Please clearly record ALL key discussion points. You will be asked to share your group insights in plenary.

**Strengths**

Questions to consider:

- What are the strengths of current Antarctic science-policy engagement?
- What are the drivers for these strengths?

**Weaknesses**

Questions to consider:

- What are the weaknesses of current Antarctic science-policy engagement?
- What are the factors influencing these weaknesses?
- How can weaknesses be overcome?

**Opportunities**

Questions to consider:

- What are the opportunities – now and into the future?
- How can strengths be used to take advantage of opportunities?
- What changes would you like to see?

**Threats/risks**

Questions to consider:

- What are the threats and barriers to Antarctic science-policy engagement?
- What internal or external factors threaten science-policy engagement?
- What can be done about the weaknesses to make the threats less likely?
- Is anything at risk if we don’t pay attention to the Antarctic science-policy interface?

## Appendix B.5. Scoring guide

The following rating scale/scoring guide was provided to participants. Three fictitious cases were provided to help give a frame of reference (Kahneman, 2021).

|  | | | | |
| --- | --- | --- | --- | --- |
| Very poor | Poor | Average | Good | Excellent |
| 1 | 2 | 3 | 4 | 5 |
| Fictitious case: Country A does not have a clear competent body that leads on Antarctic science-policy engagement. Antarctic research is only seen as important from an academic standpoint, and policymakers in Country A see no links between Antarctic research and policy (despite being an Antarctic Treaty Consultative Party). Funding for Antarctic research is largely awarded to blue skies research and research impact is judged on the basis of publications and citation indexes. There is no Government strategic direction for Antarctic research. |  | Fictitious case: Country B has a Ministry that leads Antarctic science-policy engagement. Within the Ministry there is a clear mechanism that creates a regular and ongoing dialogue between research and policy communities. The Ministry spearheads the strategic direction, which includes aligning science and policy timelines so that future needs are met. While this process is helpful, there are no incentives in place for policymakers outside of the Ministry and for Antarctic researchers to participate. Engagement is therefore dominated by the same ‘players’ who have the time, interest and connections, which has led to an increase in trust between the parties involved, but has excluded other important perspectives. |  | Fictitious case: Country C has a Ministry that leads on Antarctic science-policy engagement. The Ministry has a fully resourced science-policy engagement programme that is headed by transdisciplinary experts who guide stakeholder interactions. A clear system map clarifies the links between the Ministry and other key entities, such as research funders and academic institutions. Five-yearly horizon scanning exercises are run by the programme to anticipate future challenges and needs, and to ensure that science and policy timelines are well-aligned so that the identified needs and challenges are addressed in a timely and useful manner. The programme has internships for early career researchers and indigenous scholars to increase capacity building for future generations. |

1. During the process of developing our methods we asked ourselves whether our group discussions fell into the category of ‘breakout groups’ or ‘focus groups’ as the boundary between workshops and focus group methods are becoming increasingly blurred in the academic literature (Caretta et al., 2015). We decided to use the term ‘breakout groups’ because the group discussions that were facilitated did not entirely reflect traditional focus group characteristics such as having the primary research investigator guide the group discussion. While we had voluntary group facilitators in workshop 1, our Zoom breakout groups in workshop 2 were largely self-organising around several key tasks. We therefore did not want to muddy the water by using ‘focus groups’ as the overarching descriptor. Additionally, we see workshop breakout groups as a legitimate research method and hope that through their use we can raise the profile of workshops as a useful method for other researchers to utilise. [↑](#footnote-ref-1)
